# Impact of telenurse-led intervention in clinical trials on health literacy, empowerment, and health outcomes in patients with solid tumours: a pilot quasi-experimental study

**DOI:** 10.1186/s12912-023-01641-x

**Published:** 2024-02-02

**Authors:** Sergio Muñoz-Villaverde, María Martínez-García, Leticia Serrano-Oviedo, Francisco Javier Gómez-Romero, Ana María Sobrado-Sobrado, María Ángeles Cidoncha-Moreno, Juan Riesgo-Martín, Guillermo Pedreira-Robles, Paloma Garcimartin

**Affiliations:** 1 Oncology Clinical Trials Unit, Hospital del Mar, Barcelona, Spain; 2 Cancer Research Programme, IMIM (Hospital del Mar Medical Research Institute), Barcelona, Spain; 3 Casc Antic Primary Care Centre, Catalan Institute of Health, Barcelona Territorial Management, Barcelona, Spain; 4 Department of Medical Oncology, Hospital del Mar, Barcelona, Spain; 5 Translational Research Unit, University General Hospital of Ciudad Real, Servicio de Salud de Castilla-La Mancha (SESCAM), Ciudad Real, Spain; 6 Faculty of Medicine of Ciudad Real, University of Castilla-La Mancha, Ciudad Real, Spain; 7 ISS Bioaraba, Nursing Subdirectorate, Osakidetza General Directorate, Vitoria- Gasteiz, Álava, Spain; 8 ESIMar (Mar Nursing School), Universitat Pompeu Fabra Affiliated, Parc de Salut Mar, Barcelona, Spain; 9 SDHEd (Social Determinants and Health Education Research Group), IMIM (Hospital del Mar Medical Research Institute), Barcelona, Spain; 10 Nursing department, Hospital del Mar, Parc de Salut Mar, Barcelona, Spain; 11 Research Group in Nursing Care, IMIM (Hospital del Mar Medical Research Institute), Barcelona, Spain; 12 Biomedical Network Research Centre for Cardiovascular Diseases, CIBERCV (Carlos III Health Institute), Madrid, Spain

**Keywords:** Advanced practice nursing, Telenursing, Clinical trial, Clinical oncology, Health education, Health literacy, Empowerment, Quality of life, Patient satisfaction

## Abstract

**Background:**

During the COVID-19 pandemic, decentralised clinical trials incorporated self-monitoring, self-reporting, and telenursing tools to address health literacy and health empowerment of patients enrolled in clinical trials. We aimed to determine the impact of an educational intervention using telenursing consultations on health literacy, health empowerment, and health-related quality of life in cancer patients enrolled in clinical trials by measuring the level of satisfaction with the care received and assessing the views of healthcare professionals concerning the advanced practice nurse (APN) role in oncology clinical trials.

**Methods:**

In this pilot analytical, descriptive, longitudinal, quasi-experimental, and pre-post test study, an educational intervention was conducted by 5 visits with an APN using synchronous teleconsultation in patients starting cancer treatment for the first time in a clinical trial (*n* = 60), and health professionals working with the APN (*n* = 31). A descriptive analysis of the samples and questionnaires were utilised along with statistical comparisons.

**Results:**

After the intervention, patients' health literacy (31.7%), health empowerment (18.3%), and health-related quality of life (33.3%) increased (*p* < 0.05), with a decrease and trend towards resolution of care needs (*p* < 0.05). Satisfaction with the quality and care received in terms of perceived convenience, transition, and continuity of care showed positive results in 64.9 ± 20.7, 77.6 ± 19.5, and 72.1 ± 20.4 of respondents, respectively. On the overall assessment of the APN role, healthcare professionals expressed a high level of agreement with the statements related to their work performance.

**Conclusions:**

The data indicates that a clinical trial APN-led telenursing educational intervention results in an overall increase in health literacy, an improvement in health empowerment and health-related quality of life, and a decrease in care needs of oncology clinical trials patients. Patients stated that they received a high quality of care and health professionals indicated high levels of acceptance with APNs. Based on these results, we suggest that the APN role should gain more recognition in the Spanish healthcare system and their professional competencies should be aligned with those of other countries.

**Supplementary Information:**

The online version contains supplementary material available at 10.1186/s12912-023-01641-x.

## Background

With 935 clinical trials (CTs), Spain had the highest number of any European country in 2021. Germany (706), France (617), Italy (615), and the United Kingdom (445) followed [1]. Care of patients participating within a CT requires health professionals to act as guides in the process [2]. In certain health systems, such as in the United States, advanced practice nurses (APNs) [3–5] are charged with this responsibility. When available, APNs are considered to have a fundamental role within interdisciplinary teams since they act as consultants and personal care needs coordinators; they are a direct communication link between patient and health team; they possess a high degree of expert knowledge in specific areas of clinical care; and they possess additional skills such as leadership, communication, research, expert planning, and education. These qualities have been reported to be beneficial to the care provided [6–9].

In recent years, and reinforced by the COVID-19 pandemic [10], decentralised clinical trials (DCTs) [11] have proliferated with the aim of reducing unnecessary in-person visits. DCTs have transformed traditional practices of care by incorporating self-monitoring and self-reporting strategies and using telenursing tools [11–13] to improve health literacy (HL), patient health empowerment (HE), and health-related quality of life (HRQoL). The concept of HL emerged in the 1970s and influenced both public health and healthcare systems [14–17]. The World Health Organisation (WHO) defines it as "the social and cognitive skills that determine a person's level of motivation and ability to access, understand and use information in ways that enable them to promote and maintain good health" [18]. HE, on the other hand, was introduced in the 1960s [19, 20] and is defined by WHO as "a process by which people gain greater control over decisions and actions that affect their health" [21]. Finally, HRQoL is defined as "the perceived physical and mental health of an individual or group over time" [22]. Multiple studies have reported the benefits of APN in increasing HL, HE, and HRQoL [23].

Despite the volume of CTs performed in Spain and the benefits reported in other countries that have incorporated APNs in this field, little is known about the APN role in the field of CTs in Spain. Therefore, it is necessary to better understand the current status of advanced nursing roles in Spanish CTs and gauge whether it would be beneficial to elevate their professional competencies such that they are on a par with those of other countries [24–26]. It is important to mention that, in Spain, the APN figure is neither regulated nor accredited, although the role it is performed in some hospitals across the country following the Anglo-Saxon models of Australia, Canada, New Zealand and the United Kingdom [23–25].

Thus, the main objective of this study was to evaluate the impact of an educational intervention through a telenursing consultation on HL, HE, and HRQoL in patients enrolled in CTs with solid tumours at any stage of the disease. The second objective was to determine the opinions of health professionals regarding the APN role in oncological CTs.

## Methods

### Study design

A pilot analytical, descriptive, longitudinal, quasi-experimental, pre-post test study was conducted. No control group was included as it was understood that the aim of the intervention was to improve participants’ HL, HE, and HRQoL in accordance with literature in other contexts and settings. For the development of this study, TREND guidelines for non-randomised and quasi-experimental study designs were followed [27].

### Study population

For the main objective, the participant population consisted of patients starting cancer treatment for the first time within a CT in the medical oncology department of a tertiary hospital located in the city of Barcelona. The inclusion criteria were: a) patients over 18 years of age, b) patients with solid tumours at any stage of the disease, and c) patients who had not previously received cancer treatment within a CT. Exclusion criteria were: a) patients with no availability of remote connection devices, b) patients with instability in their clinical situation (i.e., scores 2–3 on the Eastern Cooperative Oncology Group (ECOG) functionality scale), and c) patients with cognitive impairment or a illiteracy or language barrier that prevented completion of questionnaires and following a telenursing programme.

Selection of participants was carried out from March 2021 to March 2022 using non-probabilistic, consecutive sampling until a representative sample size was obtained. For this, it was considered that if accepting an alpha risk of 0.05 and a beta risk of 0.2 in a bilateral contrast, 42 subjects would be required to detect a difference equal to or greater than 6 units in the HLS-Q12 questionnaire. A standard deviation (SD) of 12 [28–30] was assumed and estimated a loss-to-follow-up rate of 25%.

For the secondary objective, the participants were health professionals. The inclusion criteria were: a) doctors, nurses, health administrators, and psychologists from the hospital centre and the primary care centre (CAP, as for the Spanish abbreviation) in the area of Catalonia who collaborated with the APN during the course of the intervention.

### Study visits and intervention

Five fixed visits (V) were made, the intervention phase comprising V1 to V4 and a follow-up phase (V5). The first visit took place before starting treatment (V1), the second 24 h after starting treatment (V2), the third 10 days after starting treatment (V3), the fourth visit coincided with the end of the educational intervention and the start of a new treatment cycle depending on the schedule of each CT (V4), and the last visit (V5) was 3 months after inclusion. On-demand consultations were also provided.

Patients received an educational intervention with an APN via synchronous teleconsultation. Patients' digital competences were assessed with three simple questions: Do you have a mobile device such as a smartphone or tablet? Do you have an internet connection? Do you know how to perform a search on the internet? If the answer to any of the questions was NO, a follow-up phone call was made. If the answers were YES, a video call was made via the DOCTIVI® telecare application.

The educational intervention, through a semi-structured and open interview, consisted of informing the patient about the CT they were starting, clarifying doubts about the cancer disease process, providing health education for the recognition of side and adverse effects of the trial treatment, and information on alarm signs and symptoms, to both patients and their relatives. Time was given to patients and relatives to express their feelings, doubts, and expectations of their oncological situation. Health education content were based on information from the corresponding CT, informative documents, guidelines, and protocols of the centre where the study was conducted, and the responses collected from HRQoL questionnaires. Health education proportionated was variable for each patient depending on their needs.

At the beginning of the intervention, internal communication was established with principal investigators, the oncology nursing consultation, the oncology nurse continuous care consultation, and the CAP staff. This initial communication was carried out to inform professionals of patients being followed up by the CT APN, to share information between care levels, and to avoid duplication of visits.

Communication channels between care levels were established by contacting case management nurses (CMNs) in the city of Barcelona and other cities in the autonomous community of Catalonia where the patients had their CAP. Finally, CMNs communicated with the patient´s basic care team (UBA, as for the Spanish abbreviation), which included doctors, nurses, and health administrative staff, of the inclusion of a patient in a CT.

### Study variables

Sociodemographic, clinical, and psychosocial variables were collected for the main objective. Sociodemographic variables included age, gender, marital status, level of education, and whether there were cohabitants at home. Clinical variables collected were: trial phase, tumour type and stage, presence and number of comorbidities, pharmacological treatment received, adherence to treatment, self-monitoring of vital signs, independence degree, oncological symptomatology management, health education provided, COVID-19 symptomatology and COVID-19 tests performed, reminders about future clinic appointments, consultations with other professionals, need for emergency consultations between visits, level of HL, HE, and HRQoL, and satisfaction with the quality of care received (Additional file 1: Case Report Form). The following psychosocial variables were quantified: cognitive state and emotional distress.

Regarding the second objective, age, gender, job position, years worked, and the service or professional unit to which they belonged were collected, as well as the health professionals' opinions on the APN role.

### Measurements

For the main objective the following measuring tools have been used.

Comorbidities were grouped using the abbreviated Charlson index relating long-term mortality to patient comorbidity (no comorbidity: 0–1 pts; low comorbidity: 2 pts; high comorbidity > 3 pts) [31]. The use of this questionnaire has been previously validated in the typology of patients treated in this research and in the study context, demonstrating its prognostic and stratifying utility for the risk of complications [32].

The management of oncological symptomatology was quantified by recording and assessing expected outcomes and interventions according to the standardised care plan of the oncological patient needs, as well as the presence of adverse events (AEs) through the Common Terminology Criteria for Adverse Events (CTCAE). The CTCAE measures AEs from G1 (mild AE) to G5 (AE-related death) according to the established defining characteristic and helps to establish severity of AE and whether the AE is related to treatment by chemotherapeutic agents, radiotherapeutic agents, and immunotherapy.

Cognitive status was measured using Pfeiffer questionnaire [33] for screening and Mini-Mental State Examination (MMSE) questionnaire [34] in case of an abnormal result to Pfeffer questionnaire. Both questionnaires measure cognitive impairment throughout different questions and areas [33, 34].

Patient-reported outcome measures (PROMs) and patient-reported experience measures (PREMs) methodologies were used in this study [35, 36].

The following PROMs were used: To measure HL, the HLS-Q12 questionnaire was used [28]. HE was measured using the Patient Empowerment in Long term Conditions (PELC) questionnaire [20, 37] and the Health Needs Assessment (HNA) tool [38, 39] which aim to identify the individual care needs of patients. HLS-Q12, a hetero-administered 12-item scale, measures HL on a Likert scale from 1 (very difficult) to 4 (very easy). The theoretical range is from 12 to 48 points, with higher scores relating to higher HL [28]. PELC is a self-administered questionnaire that measures empowerment in chronically ill patients and contains 47 items that are scored on a Likert scale from 1 (strongly disagree) to 5 (strongly agree). The scale ranges from 47 to 235, with higher scores indicating higher levels of empowerment [20, 37]. Emotional distress and individual care needs of patients were collected using the HNA tool [38, 39] which comprises a self-assessment of health needs of patients living with cancer through a simple questionnaire. It measures the physical, practical, emotional, spiritual, social, socio-economic, and environmental needs of individuals. A higher number of marked needs indicates a higher number of concerns about the disease process [38, 39].

The PROM developed by the European Organisation for Research and Treatment of Cancer (EORTC QLQ-C30) [40, 41] and the scale designed by ECOG [42] from the United States and validated by WHO were used to assess HRQoL. The 30-item EORTC QLQ-C30 scale incorporates 5 functional dimensions (physical functioning, activities of daily living, emotional functioning, cognitive functioning, and social relationships), three symptom scales (fatigue, nausea and vomiting, and pain), a global health status scale, and several individual items to assess additional symptoms commonly reported by cancer patients. All measures range in score from 0 to 100. A high score for the functional scale represents a high/healthy level of functioning, a high score for the global health status represents a high HRQoL, but a high score for a symptom scale represents a high level of symptomatology/problems [40, 41]. ECOG is a hetero-administered scale that assesses the evolution of the patient's abilities in daily life while maintaining maximum autonomy, and results help to guide therapeutic decisions and the prognosis of the disease. The ECOG is scored from 0 to 5 (normal to death, respectively) [42].

The PREM utilised was the patient satisfaction questionnaire on the quality of care received developed by EORTC (OUT-PATSAT7) [43, 44]. EORTC OUT-PATSAT7 is a questionnaire to assess specific aspects of perceived quality of cancer care. It comprises two multiple-item scales to assess appropriateness and transition of care and one item to assess perceived continuity of care. All scales and individual item measures range in score from 0 to 100. A high score represents a high level of satisfaction with care/perceived quality of care [43, 44].

In terms of assessing the CT APN in oncology role, and to give answer to the second objective the Opinion Rating Scale of professionals who Share Health Objectives with Advanced Practice Nurses in Hospitals (EVOHIPA, as for the Spanish abbreviation) was used [45]. EVOHIPA is a tool aimed at professionals who share health objectives with hospital APNs and assesses health professionals' views on the APN role. It consists of a section on the demographic characteristics of the participants, 15 questions on the APN and 8 dimensions with 41 Likert-type response statements with 7 response options: from no answer, to 0-strongly disagree, to 5-strongly agree [45].

All study questionnaires used have their validated Spanish analogues [20, 28, 31, 33, 34, 37–45] and have been used accordingly for measuring data from Spanish-speaking patients.

### Data collection

In V1, prior to treatment start and after signing the informed consent form, data were collected on sociodemographic and clinical variables and information on HLS-Q12, PELC, EORTC QLQ-C30, and HNA instruments. In V2 and V3, clinical variables were collected, and questions were answered based on HNA results. In V4, clinical variables and HLS-Q12, PELC, EORTC QLQ-C30, HNA, and OUT-PATSAT7 questionnaires were collected. In V5, information was collected, or questions were answered according to the patients' needs and it was documented whether the patient was still on treatment or had to discontinue treatment for any reason.

Self-administered questionnaires for both patients and professionals were sent by e-mail via Microsoft Forms® with a mandatory response design. Sociodemographic and patients’ clinical data collection was performed using RedCap®.

### Data analysis

After analysing variable normality, the statistical significance of pre-post changes, defined by the educational intervention through the telenursing tool, was assessed using Pearson correlation analysis, paired measures student t-tests, and ANOVA to study the association between variables. In addition, a descriptive analysis of the characteristics of the sample was considered. For HLS-Q12, PELC, EORTC QLQ-C30, and HNA questionnaires, a descriptive analysis was performed.

Quantitative variables were described in terms of means and SD, while qualitative variables were described as numbers and percentages. Statistical packages IBM® SPSS® Statistics 23 and R software version 4.1.0 were used; statistically significant values were set to *p* < 0.05.

## Results

**Fig. 1:**
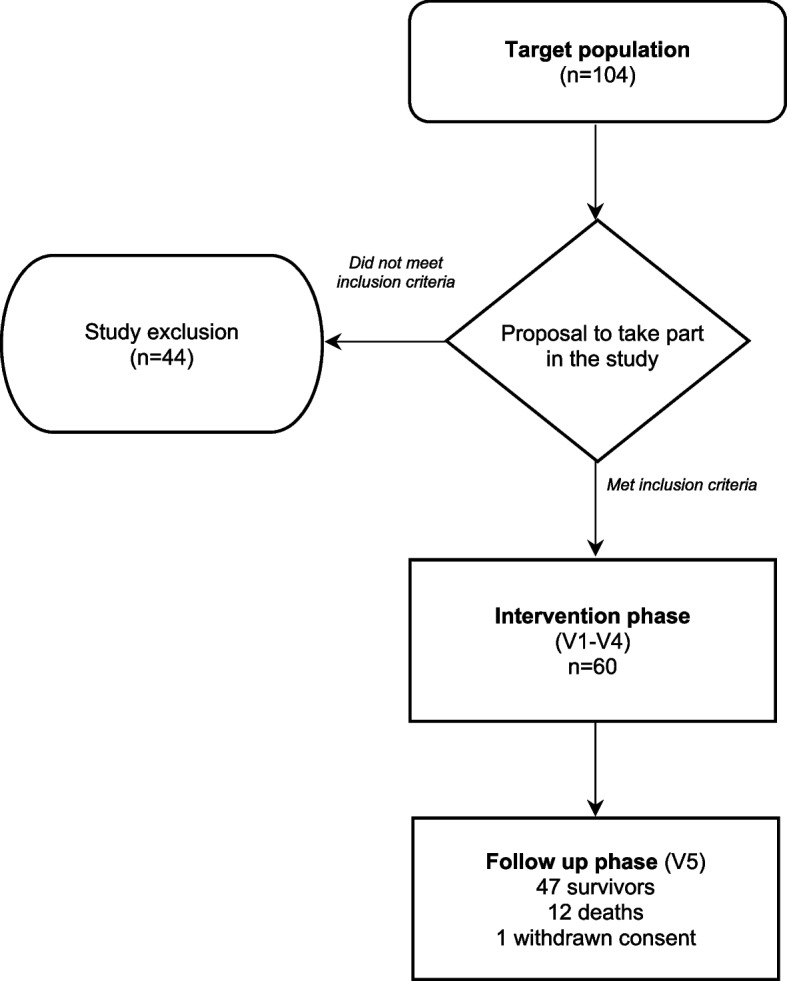
Inclusion flow chart for study participants

A total of 104 patients were selected as candidates for the study, of which 60 met the inclusion criteria. Figure 1 shows the participant inclusion flow chart and Table 1 shows their sociodemographic and clinical characteristics. Table 2 provides information on pre-post intervention changes in clinical variables. All patients had normal cognitive status as measured by the Pfeiffer mental status questionnaire and did not require a second assessment via the MMSE.

**Table 1:** Sociodemographic and clinical characteristics of the study group (*n* = 60)

Sociodemographic variable	
Age [mean years ± SD]	69.1 ± 10.4
Gender	
Female	20 (33.3%)
Male	40 (66.7%)
Marital Status	
Partner	38 (63.3%)
Single	13 (21.7%)
Widowhood	9 (15%)
Level of education	
Primary education	26 (43.3%)
Secondary education	26 (43.3%)
University degree	8 (13.3%)
Cohabitation	
Live accompanied/independent	38 (63.3%)
Live accompanied/caregiver	8 (13.3%)
Live alone/independent	14 (23.3%)
Clinical variables	
Trial phase	
Phase 1	13 (21.7%)
Phase 2	28 (46.7%)
Phase 3	19 (31.7%)
Tumour type	
Colo-rectal	6 (10%)
Esophagogastric	2 (3.3%)
Genitourinary	27 (45%)
Breast	5 (8.3%)
Otorhinolaryngology	1 (1.7%)
Lung	18 (30%)
Skin	1 (1.7%)
Tumour stage	
Stage 2	11 (18.3%)
Stage 3	6 (10%)
Stage 4	43 (71.7%)
Comorbidities	
Yes	59 (98.3%)
No	1 (1.7%)
Nº of Comorbidities (Abbreviated Charlson index)	
No comorbidity	5 (8.3%)
Low comorbidity	18 (30%)
High comorbidity	37 (61.7%)
Type of comorbidity	
Cardiovascular disease	42 (70%)
Musculoskeletal diseases	32 (53.3%)
Respiratory diseases	26 (43.3%)
Nervous system diseases	23 (38.3%)
Excretory diseases	21 (35%)
Digestive diseases	20 (33.3%)
Endocrine diseases	15 (25%)
Reproductive diseases	13 (21.7%)
Immunological diseases	10 (16.7%)
Skin diseases	5 (8.3%)
Lymphatic diseases	2 (3.3%)
Trial treatment	
Chemotherapy	11 (18.3%)
Chemo-immunotherapy	28 (46.7%)
Immunotherapy	9 (15%)
Targeted therapies	3 (5%)
Hormonotherapy	9 (15%)
Nº of patients tested against COVID-19	
Symptomatic	5 (8.3%)
Asymptomatic	55 (91.7%)
Positive COVID tests	0 (0%)
Negative COVID tests	5 (8.3%)

Values shown are n (%) unless otherwise indicated

*SD* standard deviation

**Table 2:** Pre-post intervention results of treatment adherence, self-monitoring, AE, ECOG, and symptomatology management in the study population (*n* = 60)

***Clinical variables***	
**Treatment adherence pre-intervention**	
Yes	60 (100%)
No	0 (0%)
**Treatment adherence post-intervention**	
Yes	56 (93.3%)
No	4 (6.7%)
**Vital signs self-monitoring pre-intervention**	
Yes	42 (70%)
No	18 (30%)
**Vital signs self-monitoring post-intervention**	
Yes	38 (63.3%)
No	22 (36.7%)
**AE pre-intervention**	
Yes	5 (8.3%)
No	55 (91.7%)
**AE post-intervention**	
Yes	23 (38.3%)
No	37 (61.7%)
**ECOG pre-intervention**	
0	37 (61.7%)
1	23 (38.3%)
**ECOG post-intervention**	
0	35 (59.3%)
1	17 (28.8%)
2	2 (3.4%)
3	5 (8.5%)
**Symptomatology management at the end of the intervention**	
Yes	52 (86.7%)
No	8 (13.3%)

Values shown are n (%)

*AE* adverse event, *ECOG* Eastern Cooperative Oncology Group, *SD* standard deviation

A total of 232 visits were made, 71 (30.6%) of the consultations were via the DOCTIVI® application and 161 (69.4%) via telephone along with a total of 37 on-demand visits, where 13 (35.1%) were via DOCTIVI® and 23 (64.9%) via telephone.

For psycho-emotional and spiritual assessments using the HNA tool, there were improvements compared to the initial assessments in both dimensions. 48 patients (80%) expressed emotional distress pre-intervention compared to 41 (68.6%) post-intervention (*p* < 0.001). 21 patients (35%) expressed spiritual distress pre-intervention versus 14 (23.5%) post-intervention (*p* = 0.005).

45 (75%) of the patients could be cared for autonomously. 15 (25%) patients required 93 referrals to different services and/or health professionals. The referrals were: 29 (31.1%) to the referral oncologist, 28 (30.1%) to the coordination team and CT nurses, 11 (11.8%) to continuing care nurses, 9 (9.6%) to the referral CAP, 6 (6.5%) to psycho-oncology, and 6 (6.5%) to nutrition and dietetics. Other referrals were to urology, social work, the home care programme, support teams, and oncogeriatrics, with a frequency of 1 (1.1%).

A record was kept of the need to attend to the emergency department between visits after administration of trial medication in V1. Patients who needed to be seen in one of the emergency departments (hospital emergency, continuous care, or CAP) were 1 (1.7%) between V1-V2, 7 (11.7%) between V2-V3, 11 (18.3%) between V3-V4, and 8 (13.5%) in V5.

The most common G1 AEs reported through the CTCAE v5.0 and assessed by the APN were: 20 (33.3%) fatigue, 17 (28.3%) pain, 12 (20%) nausea, 9 (15%) diarrhoea, 9 (15%) constipation, 6 (10%) anorexia, 6 (10%) headache, 5 (8.3%) dry cough, 4 (6.7%) alopecia, 4 (6.7%) urinary tract infection, 3 (5%) asthenia, 3 (5%) gastro-oesophageal reflux, 3 (5%) rash, 3 (5%) fall, 2 (3.3%) vomiting, 2 (3.3%) hot flushes, and 2 (3.3%) dysuria. Regarding the most common G2 AEs reported: 3 (5%) fatigue, 3 (5%) pain, and 2 (3.3%) alopecia. Other G1 AEs were: numbness of the mouth, anxiety, hypotension, hiccups, mucositis, facial flushing, injection site reaction, xerostomia, dizziness, dental abscess, haematuria, glans penis discomfort, dry eye, restless legs, and confusion. Other G2 AEs were: asthenia, diarrhoea, fever and confusion. All of these were reported with a frequency of 1 (1.7%).

### Results from the evaluation of questionnaires HLS-Q12, PELC, EORTC QLQ-C30 and HNA

Differences in scores of HLS-Q12, PELC, EORTC QLQ-C30, and HNA questionnaires between V1 and V4 were statistically significant (Table 3). The response rate to questionnaires in V1 was 100%. The response rate in V4 was 83.3% to the HLS-Q12 and PELC questionnaires and 85% to EORTC QLQ-C30 and HNA.

**Table 3:** Overall score changes on the HLS-Q12, PELC, EORTC QLQ-C30, and HNA questionnaires (*n* = 60)

**Questionnaires**		**Before intervention**			**After intervention**				
	**Theoretical range**	**Observed range**	**Mean ± SD**	**MV (%)**	**Observed range**	**Mean ± SD**	**MV (%)**	**Change (mean ± SD)**	***p*-value**
**HLS-Q12**	12–48	21–45	32.4 ± 5.2	0 (0)	22–48	35 ± 5.1	10 (16.7)	3.3 ± 14.5	0.025
**PELC**	47–235	109–221	161.2 ± 22.1	0 (0)	132–214	171.6 ± 20.1	10 (16.7)	18.2 ± 65.3	0.001
**EORTC QLQ-C30**									
Functional scales	0–100	10.7–100	72.2 ± 22.9	0 (0)	18–100	72.8 ± 24.0	9 (15)	-9.3 ± 31.0	< 0.001
Symptom scales	0–100	-1.2–74.7	21.6 ± 18.3	0 (0)	-1.2–58	18.3 ± 15.9	9 (15)	6.1 ± 17.6	< 0.001
Global health status	0–100	0–100	54.2 ± 22.9	0 (0)	0–91.7	55.9 ± 23.7	9 (15)	6.7 ± 29.4	< 0.001
**HNA**									
D1 + Physical concerns	0–28	0–28	9.9 ± 7.6	0 (0)	0–23	6.2 ± 5.6	9 (15)	-4.7 ± 7.0	< 0.001
D2 + Practical concerns	0–16	0–16	3.6 ± 3.9	0 (0)	0–13	2.4 ± 3.1	9 (15)	-1.5 ± 3.6	
D3 + Emotional concerns	0–12	0–12	4.2 ± 3.5	0 (0)	0–12	3.3 ± 3.5	9 (15)	-1.5 ± 3.6	
D4 + Family/relationship concerns	0–5	0–5	1.3 ± 1.6	0 (0)	0–5	1.1 ± 1.6	9 (15)	-0.4 ± 1.4	
D5 + Spiritual or religious concerns	0–3	0–2	0.4 ± 0.6	0 (0)	0–2	0.3 ± 0.5	9 (15)	-0.2 ± 0.7	
D6 + Lifestyle or information needs	0–11	0–11	4.3 ± 3.0	0 (0)	0–9	2.9 ± 2.8	9 (15)	-1.9 ± 2.9	
**TOTAL**	0–75	0–69	23.9 ± 17.2	0 (0)	0–61	16.1 ± 14.6	9 (15)	10.2 ± 15.3	

Theoretical and observed range expressed in min–max

*D* dimension, *EORTC QLQ-C30* European Organisation for Research and Treatment of Cancer Quality of Life Questionnaire—Core 30, *HLS-Q12* Health Literacy Survey-Questionnaire 12, *PELC* Patient Empowerment in Long term Conditions, *HNA* health deed assessment, *MV* missing values, *SD* standard deviation

### Results from evaluation of the EORTC OUTPATSAT7 questionnaire

Of the 60 questionnaires administered to patients in V4, a total of 51 (85%) responses were collected. Appropriateness, transition, and perceived continuity of care showed results of 64.9 ± 20.7, 77.6 ± 19.5, and 72.1 ± 20.4, respectively, indicating that, overall, satisfaction with the quality of care received was high.

### Results from the EVOHIPA questionnaire

The EVOHIPA questionnaire was distributed among 58 professionals involved in the health care process of cancer patients, and a total of 31 (53.4%) responses were collected. Sociodemographic characteristics are shown in Table 4.

**Table 4:** Health professional sample characteristics (*n* = 31)

***Sociodemographic variables***	
**Age** [mean years ± SD]	42.90 ± 10.11
**Gender**	
Female	26 (83.9%)
Male	5 (16.1%)
**Job position**	
Nurse	17(54.8%)
Doctor	10 (32.3%)
Psychologist	2 (6.5%)
CT coordinator	1 (3.2%)
Administrative	1 (3.2%)
**Years of employment**	
5 or less	4 (12.9%)
6–10	7 (22.6%)
11–20	10 (32.3%)
21 or more	10 (32.3%)
**Service or professional unit**	
CAP-UBA nurse	2 (6.4%)
CAP-UBA doctor	1 (3.2%)
CAP-CMN	9 (29%)
Hospital-CMN	1 (3.2%)
Hospital-oncology day hospital	13 (42%)
Hospital-oncology hospitalisation	1 (3.2%)
Hospital-CT oncology/haematology	4 (13%)

Values shown are n (%) unless otherwise indicated

*CAP* primary care centre, *CT* clinical trial, *CMN* case management nurse, *SD* standard deviation, *UBA* basic care team

Regarding health professionals' knowledge of the APN role, 21 (67.7%) were aware of its existence, 28 (90.3%) had previously worked with them, 20 (64.5%) received support in making a clinical decision, and 16 (51.6%) received training from the APN. Table 5 shows the results of the overall assessment of the APN role by EVOHIPA dimensions.

**Table 5:** Results by dimensions of health professional´s assessment on the clinical trial advanced practice nurse in oncology role

Variable	Mean ± SD
Dimension: Role activities	4,15 ± 0.71
Dimension: Development and teamwork	4.15 ± 0.60
Dimension: Leadership	3.98 ± 0.89
Dimension: Efficiency	4.27 ± 1.13
Dimension: Support	2.57 ± 1.36
Dimension: Recognition	3.44 ± 1.28
Dimension: Organisational model	4.61 ± 0.57
Dimension: Regulation	4.03 ± 1.14

*SD* standard deviation

## Discussion

The results of this study support the positive impact of an educational intervention led by an APN through telenursing consultation on HL, HE, and HRQoL in patients with solid tumours included in CTs. At the same time, it provides information on patient satisfaction with the care received and the opinions of healthcare professionals with the CT APN in oncology. Specifically, this study shows how an educational intervention increased HL, HE, and HRQoL in 31.7%, 18.3%, and 33.3% of patients, respectively. Reductions in expressed and felt health needs related to physical, practical, emotional, spiritual, spiritual, social, socio-economic, and environmental concerns were achieved, and those identified needs that could not be resolved by the APN were referred to the appropriate professionals. When HL and HE improvement interventions are carried out, health problems are reduced and/or better self-management of health problems occurs [46–49]. Patients expressed high satisfaction with the quality of care received, as reported in the literature by using PROMs and PREMs the quality of care can be increased [50–52]. In terms of the overall assessment of the CT APN in oncology role, healthcare professionals indicated high levels of acceptance of the role [53] within the interdisciplinary team.

Studies that have measured the effectiveness of educational interventions using audio-visual material and/or educational guides in face-to-face format or with telenursing tools to assess the HL and/or HE of patients with cancer and other chronic diseases have shown an increase in HL and HE [15, 37, 49, 54, 55]. This indicates that such educational interventions have a positive impact on the level of HL and HE.

The use of telenursing has been shown to help increase HL and HE in cancer patients [54, 55]. On the other hand, no differences have been found for HRQoL when comparing telenursing and face-to-face interventions [54]. This study does not have a control group, and therefore the results are not comparable; however, it does show an increase in HRQoL after the intervention (Table 2), in agreement with other studies that have assessed the role of APNs [56–59]. Other educational interventions led by non-APN have also increased HRQOL [14, 47], however, in addition to patient satisfaction APNs have the added benefit of acting as a reference professional in patient care and coordinator of interdisciplinary teams [60]. APNs make valuable contributions towards increasing safety and selecting the most appropriate clinical decisions for each situation as well as ensuring these choices are based on the best available scientific evidence [53, 61].

In other countries, nurses are often the first point of entry for identifying, linking, and treating AEs in patients included in CTs [6, 8]. The early identification of AEs by nurses is relevant for the well-being and safety of the patient and for the development of the study [8]. Additionally, APNs facilitate the transfer of information and initiate steps so the patient is referred, when necessary, to the relevant professional who can respond to an AE. There have been calls for nurses to occupy leadership positions within CTs, such as sub-investigators [62]. The increase in the competencies and training of APNs is generating new models of clinical research leading to their positive incorporation in CTs [62].

Findings on treatment adherence, self-monitoring of vital signs, AE and ECOG have shown that after the intervention some of the patients worsen in these items, our theory is that this could be due to the complex aetiology of cancer affecting their performance differently depending on the stage or severity of the oncological disease [46, 63, 64].

In terms of healthcare professional assessment on the CT APN in oncology role, the results of the EVOHIPA scale showed that the CT APN in oncology role has high levels of acceptance within the interdisciplinary team; these data are comparable to other national and international studies where the importance of the APN has been highlighted [45, 53, 65, 66]. In our study, health professionals of the CAP (CMN and UBA) expressed a very positive relationship between CAP and hospital, emphasising the importance and need for good communication between both areas for correct management of oncological CT patient symptomatology, which is important since many other studies have indicated a lack of communication between primary and hospital care [67, 68].

### Limitations

This pilot study has limitations due to its methodological conception. Firstly, educational intervention follow-up times were variable and dependent upon the CT, which may have had an impact on the measurement of patients' HL, HE, and HRQoL. In addition, a representative but small and heterogeneous sample has been included in this pilot study. Variables such as age, sex, disease stage, type of treatment, study phase and/or ECOG have been studied, which have not allowed a multivariate statistical analysis to stratify the collected variables. This could potentially have weakened the external validity of the study. Despite this, benefits of the analysed intervention have been shown, and in possible future studies we will consider a larger, multicentre sample size to increase the validity of the analysed intervention, through the role of the advanced practice nurse. On the other hand, although the response rate of healthcare professionals to the EVOHIPA questionnaire was over 50%, it would have been more representative to have had a higher number of responses.

## Conclusions

The results showed that an APN-led educational intervention resulted in an overall increase in HL, an improvement in HE and HRQoL, and a decrease in care needs of patients participating in oncological CTs. Patients expressed a high quality of care received and health professionals indicated high levels of acceptance of the APN role. It is important to emphasise that telenursing tools favour faster and more accessible communication with patients and health professionals involved in CTs, increasing safety.

There have been few oncological studies led by nurses where interventions were aimed at patients with different types of cancer [69], and this study adds to this scientific base of knowledge. This study is among the first to evaluate the role of the APN and telenursing in oncology CTs in Spain. As the European country that performs the most CTs, our data suggests a benefit for promoting the CT APN role in Spanish CT units. This study should be regarded as a first approach to the creation of this role and to place the professional competencies of APNs on a par with those of nurses in other countries such as the United Kingdom, the United States, and Canada. The contribution that APNs make to the healthcare system should be recognised at the national level.

### Supplementary Information

**Additional file 1**. This section presents the case report form.

## Data Availability

The datasets used and/or analysed during the current study are available from the corresponding author upon reasonable request.

## Abbreviations

AE: Adverse Event
ANOVA: Analysis Of Variance
APN: Advanced Practice Nurse
CAP: Primary Care Centre
CMN: Case Management Nurse
CT: Clinical Trial
CTCAE: Common Terminology Criteria for Adverse Event
DCT: Decentralised Clinical Trial
ECOG: Eastern Cooperative Oncology Group
EORTC QLQ-C30: European Organisation for Research and Treatment of Cancer Quality of Life Questionnaire - Core 30
EVOHIPA: Opinion Rating Scale of Professionals who Share Health Objectives with Advanced Practice Nurses in Hospitals
G: Grade
HE: Health Empowerment
HL: Health Literacy
HLS-Q12: Health Literacy Survey - Questionnaire 12
HNA: Health Needs Assessment
HRQoL: Health-Related Quality Of Life
MMSE: Mini-Mental State Examination
PELC: Patient Empowerment in Long Term Conditions
PREM: Patient-Reported Experience Measure
PROM: Patient-Reported Outcome Measure
SD: Standard Deviation
UBA: Basic Care Team
V: Visit
WHO: World Health Organization
