# Supplementary material for: Impact of telenurse-led intervention in clinical trials on health literacy, empowerment, and health outcomes in patients with solid tumours: a pilot quasi-experimental study
Source: BMC Nurs. 2024 Feb 2;23:86. doi: 10.1186/s12912-023-01641-x (PMC10835870; doi:10.1186/s12912-023-01641-x)
Supplement: Supplementary file 1 — Additional file 1. This section presents the case report form. [file 12912_2023_1641_MOESM1_ESM.docx]

**Additional file 1**

This section presents the case report form.

**SOCIODEMOGRAPHIC DATA**

- Date: ________
- Signed IC: Yes No
- Patient name: __________
- Date of birth: _______
- Gender: male, female, non-binary (I do not identify with my gender), prefer not to say.
- Marital status: partner, single, divorced, widowhood.
- Level of education: cannot read/write, primary education, secondary education, university degree.
- Cohabitation at home: live alone, live accompanied/independent, live accompanied/caregiver.

**PRIOR INITIATION OF TREATMENT (PRE C1D1)**

- Tumour type: Lung/ Breast/ Genitourinary/ Gynaecological/ Biliopancreatic/ Esophagogastric/ CNS and brain metastases/ Colo-rectal/ Otorhinolaryngology/Skin.
- Tumour stage: 0 1 2 3 4
- Trial medication: Intravenous chemotherapy/ Oral chemotherapy/ Radiotherapy/ Immunotherapy/ Oral hormone therapy/ Intramuscular hormone therapy/ Qumio-radio/ Chemo-immuno/ Radio-immuno.
- Duration of treatment: until progression/until toxicity/until clinical trial closure/time-limited trial.
- Trial phase: 1 2 3
- Comorbidities: nervous system/ endocrine/ cardiovascular/ digestive/ respiratory/ excretory/ reproductive/ musculoskeletal/ immune/ lymphatic/ integumentary system.
- ECOG: 0 1 2 3 4 5
- Cognitive status: administer Pfeiffer (if Pfeiffer positive, administer MMSE).
- Complete questionnaires: HLS-Q12, PELC, QLQ-C30, HNA.
- Contact each patient's CAP case manager to inform about inclusion in clinical trial: Yes No
- AEs/oncological symptomatology (specify and grade following CTCAE v5. 0-indicate date of onset, duration, amount and whether related to treatment or oncological disease): Anorexia/ Vomiting/ Nausea/ Xerostomia/ Mucositis/ Diarrhoea/ Constipation/ Pain (if pain, specify location and administer categorical pain scale) / Fatigue (if fatigue, administer numerical fatigue scale)/ Alopecia/ Pruritus/ Rash/ Infection/ Pneumonitis/ Fever/ Neuropathy/ Headache/ Liver alterations/ Hormonal alterations/ Renal alterations/ Other AEs. * Manage symptomatology through Hospital Health Education guidelines.
- COVID19 symptomatology ( test for Covid19 if positive for symptomatology): Does not refer/ Has been in contact with a COVID19 patient/ Fever (more than 37.5)/ Fever (less than 37.5) for more than 3 days/ Dry cough/ productive cough/ Headache/ Myalgia/ Arthralgia/ Dyspnoea/ Tiredness/ Nausea/ Diarrhoea/ Vomiting/ Skin lesions/ Chest pain/ Anosmia/ Dysgeusia/ ageusia.
- Covid19 test result: Positive/negative.
- Concomitant medication (indicate start/end date, dosage): Yes No (antihypertensive/ antiarrhythmic/ antilipidemics/ anticoagulants/ analgesia/ antacids/ anticonvulsants/ hypoglycaemics/ anti-infectives/ antiemetics/ antiallergics/ antitussives/ mucolytic/ steroids/ antidiarrheal/ laxatives/ vitamins/ mineral supplements/ food supplements/ hormonal treatments/ creams (dermatological treatments) / antidepressants/ anxiolytics.
- Self-monitored vital signs: BP/ Pulse/ RF/ Temperature/ Oxygen/ Weight.

**24 h POST C1D1 (C1D2), 10 DAYS POST C1D1 (C1D11), PREVIOUS DAY C2D1, CONSULTATION ON REQUEST***

- Date: _____
- Provide health education in all consultations regarding needs identified through the HNA questionnaire.
- ECOG: 0 1 2 3 4 5
- AEs/oncological symptomatology (specify and grade following CTCAE v5. 0-indicate date of onset, duration, amount and whether related to treatment or oncological disease): Anorexia/ Vomiting/ Nausea/ Xerostomia/ Mucositis/ Diarrhoea/ Constipation/ Pain (if pain, specify location and administer categorical pain scale) / Fatigue (if fatigue, administer numerical fatigue scale)/ Alopecia/ Pruritus/ Rash/ Infection/ Pneumonitis/ Fever/ Neuropathy/ Headache/ Liver alterations/ Hormonal alterations/ Renal alterations/ Other AEs.
- Symptomatology management: Yes No * Manage symptomatology through Hospital Health Education guidelines.
- COVID19 symptomatology (test for Covid19 if positive for symptomatology): Does not refer/ Has been in contact with a COVID19 patient/ Fever (more than 37.5)/ Fever (less than 37.5) for more than 3 days/ Dry cough/ productive cough/ Headache/ Myalgia/ Arthralgia/ Dyspnoea/ Tiredness/ Nausea/ Diarrhoea/ Vomiting/ Skin lesions/ Chest pain/ Anosmia/ Dysgeusia/ ageusia.
- Covid19 test result: Positive/negative.
- Changes in concomitant medication (indicate start/end date, dosage): Si No
- Concomitant medication (indicate start/end date, dosage): Yes No (antihypertensive/ antiarrhythmic/ antilipidemics/ anticoagulants/ analgesia/ antacids/ anticonvulsants/ hypoglycaemics/ anti-infectives/ antiemetics/ antiallergics/ antitussives/ mucolytic/ steroids/ antidiarrheal/ laxatives/ vitamins/ mineral supplements/ food supplements/ hormonal treatments/ creams (dermatological treatments) / antidepressants/ anxiolytics.
- Self-monitored vital signs: BP/ Pulse/ RF/ Temperature/ Oxygen/ Weight.
- Have you been to an emergency department since our last teleconsultation? Yes No Which one? Hospital Accident and Emergency department / GP Accident and Emergency department / Hospital centre for oncological emergencies.
- Clinical consultations/referrals (refer if necessary): trial team (nursing or coordination team)/ Hospital centre for oncological emergencies/ Accident and Emergency department/ reference oncologist / Palliative care team/ Social worker/ Psycho-oncology/ Nutritionist-Dietician/ GP for control of chronic pathologies, wound cure...
- Check adherence to oncological treatment (if oral treatment): identify risk factors in case of non-adherence.
- Reminder of future visits.

*** *On-demand consultations will solve health problems related to the reason for the consultation.*

**POST-INTERVENTION EVALUATION**

- HLS-Q12, PELC, EORTC QLQ-C30, OUT-PATSAT7 questionnaires will be administered.
- HNA will be re-evaluated.

**FOLLOW UP VISIT (3 months after the post-intervention evaluation visit)**

- Date: _____
- Patient continues trial 3 months after intervention completion: Yes No, if not: reason for ending the trial: toxicity/disease progression/clinical trial closure/treatment completion/death.
